# Building capacity for dementia care in Latin America and the Caribbean

**DOI:** 10.1590/S1980-57642014DN84000002

**Published:** 2014

**Authors:** Francisco J Gonzalez, Ciro Gaona, Marialcira Quintero, Carlos A Chavez, Joyce Selga, Gladys E Maestre

**Affiliations:** 1 Fundación Alzheimer de Venezuela, Nueva Esparta Chapter, Porlamar, Venezuela; 2 Fundación Alzheimer de Venezuela, Caracas, Venezuela; 3 Neurosciences Laboratory, Faculty of Medicine, University of Zulia, Maracaibo, Venezuela; 4 Dept. Psychiatry, Neurology and G.H. Sergievsky Center, Columbia University, New York, NY, USA

**Keywords:** health care, dementia, capacity building, Alzheimer's disease, health programs, skills, community

## Abstract

Latin America and the Caribbean (LAC) have limited facilities and professionals trained to diagnose, treat, and support people with dementia and other forms of cognitive impairment. The situation for people with dementia is poor, and worsening as the proportion of elderly in the general population is rapidly expanding. We reviewed existing initiatives and provided examples of actions taken to build capacity and improve the effectiveness of individuals, organizations, and national systems that provide treatment and support for people with dementia and their caregivers. Regional barriers to capacity building and the importance of public engagement are highlighted. Existing programs need to disseminate their objectives, accomplishments, limitations, and overall lessons learned in order to gain greater recognition of the need for capacity-building programs.

## Abstract (pt)

América Latina e Caribe (ALC) têm instalações e profissionais treinados para diagnosticar, tratar e apoiar as pessoas com demência e outras formas de comprometimento cognitivo limitado. A situação para as pessoas com demência é pobre, e piora quando a proporção de idosos na população em geral está se expandindo rapidamente. Revisamos as iniciativas já existentes, com exemplos de medidas tomadas para fortalecer a capacidade e melhorar a eficácia dos indivíduos, organizações e sistemas nacionais que fornecem tratamento e apoio às pessoas com demência e seus cuidadores. As barreiras regionais ao reforço das capacidades e a importância do engajamento público são realçados. Os programas existentes precisam divulgar seus objetivos, realizações, limitações e lições globais aprendidas a fim de obter maior reconhecimento da necessidade de programas de capacitação.

## INTRODUCTION

By 2040, 71% of the 81.1 million dementia cases worldwide will be located in the developing world.^1^ Although studies in Asia and Africa have suggested that the prevalence and incidence of dementia in Latin America and the Caribbean (LAC) would be lower than in the developed world,^1,2^ recent data show high rates of dementia in LAC, with 6-8% prevalence in subjects over 60 years of age.^1,3,4^ The estimated two million people with Alzheimer's disease and related disorders constitute a hidden epidemic in the region, rather than a group of uncommon syndromes, as once assumed.^5^ Low levels of education, high rates of brain injury, poor diet, sedentary lifestyle, and high risk of cardiovascular diseases and diabetes are among the main risk factors behind this trend. Furthermore, large family groups in the region are afflicted with dementia, including those found in the Dominican Republic, Puerto Rico, Colombia, and Venezuela.^3,5^

Despite the large numbers of people affected, LAC has limited facilities and professionals trained to diagnose, treat, and support people with dementia and other forms of cognitive impairment. The situation is worsening as the proportion of elderly in the general population is rapidly expanding.^6-10^ People with dementia are frequent users of healthcare and long-term care services.^9^ However, their primary caregivers are often unpaid family members, friends, or neighbors,^11^ who face times of stress, pain, fear, frustration, and depression. Thus, both patients and caregivers suffer from insufficient availability of medical care and social programs.

This article reviews strategies for building capacity in LAC to diagnose, treat, and support people with dementia. We include approaches that have been developed and implemented or are still in the design stage. Our goal is to better understand factors that act as barriers to or facilitate the ability of LAC countries to anticipate and respond to the public health threat of dementia.

## CAPACITY BUILDING

We use the term "capacity building" in reference to interventions that produce sustained change at individual to national levels.^12^ Strategies for capacity building can result from careful planning or as a spontaneous response to pressing needs. They can be simple and limited in time, or complex, new or already embedded in the local culture. Our analysis uses the framework of Crisp et al.,^13^ which characterizes approaches to capacity building into four categories:

(i) top-down organizational approaches;
(ii) bottom-up organizational approaches;
(iii) partnerships; and
(iv) community organizing approaches.

**Top-down organizational approaches.** Changing public policies is among the most effective mechanisms for changing conditions in society,^14-17^ and therefore, is a frequent approach of advocacy groups, outreach activities, and international health agencies.^18,19^ Public policies can set minimum standards of care, influence availability of drugs, create and sustain referral centers, and allocate resources to train healthcare personnel in remote areas or underserved communities.^2,20^

In LAC, policy changes are usually the result of strong social and political demands. The mechanisms to facilitate/accelerate changes converge in empowering a central body, such as Congress or the Ministry of Health, to take an overall view of priorities and societal gain. For example, Peru, Bolivia and Mexico have approved national plans, while Chile, Argentina, Venezuela, and Uruguay are working towards similar plans to provide timely diagnosis, treatment, and protection of Alzheimer patients.^21,22^ In a less centralized top-down approach, many advocacy groups in LAC, such as Alzheimer's associations, are part of national or international networks,^23^ and follow the guidelines and recommendations of these networks, which are then adopted and carried out by local chapters.

**Bottom-up organizational approaches.** There is still widespread belief in LAC that detrimental changes in personality and behavior are a natural result of aging.^24^ Therefore, many dementia sufferers and caregivers do not seek medical attention and support unless the symptoms are severe, and their specific problems and needs are often invisible to healthcare providers and policymakers.^25,26^ Training programs for healthcare personnel and educational programs for individuals at risk or in early stages of dementia, and their caregivers, are powerful strategies for increasing awareness and early diagnosis skills. One result is that the services and resources available in the community become more closely matched to the local burden of dementia. The encouragement of healthcare providers to become "reflective practitioners" - bringing together theory and practice through reflection - has been identified as a particularly important strategy for capacity building. ^13,27^

In LAC, the bottom-up approach has resulted in partnerships between advocacy groups, clinicians, and academic centers, which deliver training programs for healthcare professionals. For example, the Nueva Esparta Chapter of the Venezuelan Alzheimer's Foundation (Fundación Alzheimer de Venezuela), the University of Zulia, the Experimental University of Liberator, and the Ministry of Higher Education, collaborate in offering the Certificate for Comprehensive Care for Persons with Cognitive Impairment through a national scholarship program, FUNDAYACUCHO.^28^ The certificate requires 23 credits, including 98 hours of classroom instruction, and 90 hours of hands-on team projects and case studies. Of the 33 professionals initially admitted (76% women; most 40-60 years of age), 28 graduated successfully. The graduates have developed interdisciplinary work teams to establish community projects in the State of Nueva Esparta, and have created a professional support network and resources for the national Alzheimer's Foundation. To date, the community projects have provided assistance to around 1500 individuals and various educational materials have been produced to support learning.^29,30^

Education of dementia patients, high risk individuals, family members, and caregivers can facilitate the timely seeking of health care and support, but can also clarify the rights of patients and caregivers to various services, including access to medication and caregiver support. For example, the School of Caregivers of Older Adults in Maracaibo, Venezuela, provides education to non-professional caregivers of older adults, with or without dementia, residing at home.^31^ During 32 academic hours, a team of healthcare professionals acts as facilitators of peer-driven discussions and other interactions for groups of 30 participants, covering basic needs, such as hygiene, comfort, mobility, sleep, safety, nutrition, social intervention, cognitive and physical stimulation, and handling of difficult situations. Several activities are devoted to the wellbeing of the caregivers. One outcome was a written educational guide for caregivers.^32^

Each person with dementia experiences symptoms differently. The bottom-up approach allows health care and services to be flexible and address the specific needs of each patient and their caregivers. In LAC, advocacy groups have developed workshops offering physical, social, and/or cognitive stimulation. The workshops, often led by expert caregivers supported by health professionals, allow dementia patients to participate in activities such as dancing, singing, storytelling, painting, physical exercise, and formal cognitive exercises. In Maracaibo, such workshops have been held for more than 17 years,^33^ and have generated a manual of cognitive exercises that allows implementation by community members.^34^

**Partnerships approach.** Partnerships bring together individuals and/or organizations with a common interest: improving the lives of people with dementia and their caregivers, promoting early diagnosis, facilitating access to services and medication, promoting educational programs, or carrying out awareness events. In optimal situations, the partnership involves representatives of many levels: patients and caregivers, advocacy groups, academic centers, private industry, and policymakers.^35,36^ In LAC, the partnerships focused on dementia are often loosely organized. For example, an advocacy group might obtain financial support from the local Chamber of Commerce and retail stores as sponsors for a public event, such as a walk or a lecture series, but do not articulate a long-term vision for the partnership. Other types of partnerships important for developing healthcare infrastructure are those that include researchers, such as the 10/66 Dementia Research Group,^37^ and the Maracaibo Aging Study.^38^ The researchers often reside in LAC or are among those who have joined the diaspora of scientists from LAC to developed countries.^39^ However, the reasons for the dearth of formal partnerships need to be explored. The problem might be the lack of successful precedents, or previous partnerships that had difficulties. Participation by patients might be perceived as irrelevant or even counterproductive. Understaffing of advocacy and research groups might make initiating a new set of activities difficult. And disadvantaged communities often lack the resources to reach out to stakeholders and establish partnerships.^40^

**Community organizing approach.** Working at the community level, to transform individuals from passive recipients of services to active participants, is probably the most ambitious approach to capacity building.^13^ Community members must be educated in order to recognize high risk situations, caregiving needs, and signs indicating the need for professional help. They must be given opportunities to learn skills in leadership, decision-making, and conflict resolution, to develop norms and procedures, and to articulate shared visions.^41^

The federal governments of several LAC countries have promoted community participation in healthcare decisions during the last decade,^42-45^ some focused on the elderly, but none are aimed specifically at people with dementia. However, social support networks that have been developed for older persons^46-48^ and were conceived as "informal systems" that bring together individuals, peers, and organizations,^46^ might serve as starting points for the formation of support networks for dementia patients and caregivers. For example, non-governmental organizations (NGOs) and non-profit organizations in Chile have developed clubs for the elderly, associations for retirees, and hospices.^47^

## DETERMINANTS OF CAPACITY BUILDING FOR DEMENTIA CARE

Dementia puts individuals and families in a vulnerable position, with constantly emerging needs in terms of diagnosis, treatment, care, and even prevention. Even countries with highly developed societal resources struggle with the emerging needs of dementia patients and their caregivers.^48^ LAC countries have a plethora of unmet needs and, therefore, of opportunities for building capacity to deal with dementia at the individual, community, organizational, and national levels.^13,49-51^ However, capacity building is an investment in sustainable long-term improvement, which often progresses slowly, even under the best conditions.^52,53^ Building capacity depends on a series of critical system characteristics related to both physical elements (infrastructure, material wealth, technology), as well as social (human capital, political legitimacy, and institutional strength).^54^

## BARRIERS TO CAPACITY BUILDING FOR DEMENTIA CARE IN LATIN AMERICA AND THE CARIBBEAN

Most of the healthcare infrastructure in LAC has largely focused on the prevention and treatment of communicable diseases such as dengue, tuberculosis, malaria, and HIV/AIDS.^55^ Capacity building aimed at non-communicable diseases has only recently been initiated, and has focused primarily on cancer, cardiovascular diseases, and diabetes.^56^ The region is highly heterogeneous,^57^ and successful strategies for building services and research capacity will vary from country to country, even among regions within each country. However, some barriers to capacity building are common throughout LAC.

**Poverty.** Insufficient resources and wealth inequity affect capacity building at many levels. First, there are many health and social needs competing for limited funds. Second, the working and living conditions of many clinical and research staff members are less than optimal, limiting the uptake of new tasks and tools. Finally, there is unequal distribution of healthcare and related services, particularly due to the geographic dispersion of rural communities. For example, many health education programs in LAC initially focused on schools in elite urban centers.^58^ Although health and health education programs have now shifted towards the poor and disadvantaged,^59^ healthy lifestyle programs, particularly for the elderly,^60^ remain uncommon in poor, rural areas. For instance, older adults residing in rural areas of Mexico have significantly lower pensions, access to health services, and access to recreational activities than those in urban areas,^61^ and this is probably the case in other countries.

**Unfavorable political environments.** Many LAC countries are highly polarized politically, lack continuity in their ruling governments, or are plagued by perceptions of politicians as corrupt,^62^ making the political environment difficult for advocacy groups attempting to influence public policy. For example, in many countries of the region, access to coverage by the media is highly biased by political views.^63^ Furthermore, crime rates in the region are high (e.g., homicides with firearms occur at three times global average rates), creating an atmosphere of suspicion and distrust that results in generally weak institutions and poor cooperation.^64^

**Problems with existing healthcare systems.** Healthcare systems in many regions of LAC are overloaded, fragmented, and poorly coordinated, with deteriorating facilities.^65^ Subsystems with different modes of financing, affiliation and healthcare delivery serve different groups, defined by social status and the ability to pay.^66^ The common model of care is centered on episodic care of acute disease and hospital-based treatment.^59^ Continuity of care, with planned visits and regular follow up, is scarce. The existing model of health care is not appropriate for the diagnosis, treatment, and ongoing care of dementia patients.

**Insufficient information technology infrastructure.** Access to the medical and scientific literature is heterogeneous, but generally insufficient, in LAC. Educational institutions often cannot afford to maintain electronic journals, digital archives, bibliographic databases, even the printed literature, and there is no open source of reference material for chronic non-communicable disorders or geriatric medicine, as there is for tropical medicine.^67^ Furthermore, internet and email access are unreliable, and high costs of cellphone services limit communication and online learning options.^68^ Limited access to new information about the diagnosis and treatment of dementia patients, and limited ability to communicate that information to healthcare professionals and caregivers, are barriers to capacity building.

**Lack of standardized evaluation of capacity building initiatives.** Several models have been developed for monitoring and evaluating capacity building in developing countries, including capacity building in health care.^69^ However, most of these models have not been translated into Spanish or Portuguese, and there are no reports of adaptation to or testing of these models in LAC. Standardized indicators of impact are urgently needed to determine the effectiveness of current and future capacity-building initiatives.

**Insufficient mentorship.** Advocacy groups, researchers, and healthcare professionals dealing with dementia patients and caregivers benefit from the guidance and support of individuals with relevant experience. Not only do research and healthcare institutions in LAC lack a sufficient number of qualified mentors, but there is insufficient funding to involve the mentors that are potentially available. Furthermore, the role of mentor is not given the recognition that might encourage and facilitate such actions.

## FACILITATING CAPACITY BUILDING FOR DEMENTIA CARE IN LAC

Based on this review of the literature and our collective experience, we offer six recommendations for capacity-building initiatives seeking to improve the health care and services available to dementia patients and their caregivers.

1. Determine the scope of capacity building that is appropriate for the goals of the initiative.
2. Encourage participation at all appropriate levels. Develop an identity that enables cohesion among individuals, communities, social levels, organizations, and governmental agencies.
3. Develop, document, disseminate, and evaluate strategies, tools, and interventions that address specific challenges, but are adaptable to different or changing conditions.
4. Prioritize communication among patients, patient advocates, healthcare providers, researchers, and policymakers. All participants should understand the goals, processes, and time lines of the initiative.
5. Utilize all relevant healthcare resources, including geriatrics, neurology, psychiatry, family medicine, internal medicine, services for chronic non-communicable disorders, services for mental and intellectual disabilities, and rehabilitation services. Also involve resources aimed at primary care and promotion of healthy lifestyles.
6. Develop formal coalitions among advocacy groups, academic medical centers, professional societies, and governmental agencies, within the region, country, and international community.

## PUBLIC ENGAGEMENT AS A CATALYST OF CAPACITY BUILDING

Community engagement is the process of collaboration among groups of people with something in common - geographical proximity, special interests, being affected in a common way, etc.^70^ The engagement must be meaningful and have a purpose, whether implicit or explicit, that strengthens the relationship between the public and the desired impact. In the case of dementia, public engagement usually involves educating patients, their families, caregivers, and the broader community about cognitive impairment and its effects at personal, organizational, and societal levels. This is challenging in many parts of LAC, due to generally low levels of health literacy, poverty that increases the urgency of competing needs, and lack of open discussion about dementia among peers, healthcare providers, and advocacy groups. Furthermore, there are often no financial or human resources for implementing and sustaining community engagement, particularly in rural areas.

Despite the challenges, several groups in LAC have successfully involved communities in dementia issues through public events. For example, the INECO Foundation in Argentina has a program of guided visits to museums for people with dementia (www.arteycerebro.com.ar). Another example is the Interdisciplinary Symposium on Alzheimer's disease and related disorders, which has been held annually for seventeen consecutive years in Maracaibo, Venezuela (www.simposioalzheimer.com). The leaders of this event are healthcare providers and researchers, who developed a plan to educate the public about the origins of dementia, signs and symptoms, risk factors, diagnostic strategies, treatment options, and caregiver issues. They have involved advocacy groups, the media, local educators, and other interested parties. The focus is on improving the quality of life for patients and caregivers, and effective actions to allow a life with dignity.^71^ People with dementia are also encouraged to participate in group activities, such as dances, plays, concerts, and exercise routines, that demonstrate their potential for action, creativity, and emotional development. An important lesson from these experiences is that creativity and emotional rapport among presenters and participants are crucial for the success of such activities; strict presentation of scientific facts is much less effective.

Based on the successful experience of the symposium in Maracaibo, we can recommend ways to facilitate the process of community engagement.

1. Clearly define the audience, the purpose of the interaction, and the desired outcome. These will dictate the communication strategy. Even if the event has more than one purpose, e.g., educating the public, recruiting volunteers, raising money, the main purpose must remain clear.
2. Do not use medical or academic jargon, or adopt the tone of a lecturer. Be genuine and use a conversational tone. For example, one of us (CG) has used the term "our own garden" to explain the term cognitive reserve, and explained that each individual is responsible for nurturing and protecting their own garden, enabling it to survive adverse events.
3. Maintain a positive attitude. Even though there are no specific preventive measures for dementia, lifestyle modifications that reduce cardiovascular risk factors and depression/isolation can be helpful. It is useful to suggest positive actions, e.g., that people watch their weight, blood pressure, and other indicators of physical health, and that they also cultivate healthy social relationships. These recommendations are valid for people of all ages. The notion that the brain is plastic, and can be protected to some extent by a healthy lifestyle and positive mood, can inspire and empower individuals to generate positive changes in their community.
4. Incorporate local references, including humor. Latin America has a strong tradition of storytelling; local legends and traditional stories can be used to convey complex messages. Visual images should also be designed to facilitate connection with the audience.
5. Be aware of diversity. The general public is heterogeneous and will bring different levels of interest and knowledge to the group.
6. Promote action at individual, organizational, and national levels. Avoid blaming problematic situations on lack of involvement or support at any level.

## Conclusions.

There is a high level of unattended suffering among people with dementia in Latin America and the Caribbean, as well as their families and caregivers. At this point, there is insufficient capacity to respond to this suffering, because financial and logistical needs are not being adequately addressed. However, it is time to stop blaming social and political problems, and take positive steps toward capacity building. This means moving beyond rhetoric into action, and involving a broad range of stakeholders, including policymakers, researchers, clinicians, the media, and most of all, dementia patients, their families and caregivers. Evidence-informed guidelines for dementia care at the country level need to be generated and supported by the allocation of the resources required, particularly in areas where there is a higher number or density of cases. Capacity building for dementia care differs in some aspects, but can benefit from successful capacity building for other public health problems. Inter-programmatic interventions integrating various disciplines should be established. Public engagement is vital, and multilevel approaches are required to make actions sustainable. Each initiative must gain recognition by disseminating information about its objectives, accomplishments, impacts, limitations, and overall lessons. Effective and long-lasting capacity building in dementia care could impact other important aspects of society^72^ and therefore serve as a powerful adjuvant for development.
